# Using iMCFA to Perform the CFA, Multilevel CFA, and Maximum Model for Analyzing Complex Survey Data

**DOI:** 10.3389/fpsyg.2018.00251

**Published:** 2018-03-13

**Authors:** Jiun-Yu Wu, Yuan-Hsuan Lee, John J. H. Lin

**Affiliations:** ^1^Institute of Education & Center for Teacher Education, National Chiao Tung University, Hsinchu, Taiwan; ^2^Department of Education and Learning Technology, National Tsing Hua University, Hsinchu, Taiwan; ^3^Office of Institutional Research, National Central University, Taoyuan, Taiwan

**Keywords:** multilevel structural equation modeling, confirmatory factor analysis, complex survey data, Lisrel, Mplus, maximum model

## Abstract

To construct CFA, MCFA, and maximum MCFA with LISREL v.8 and below, we provide iMCFA (integrated Multilevel Confirmatory Analysis) to examine the potential multilevel factorial structure in the complex survey data. Modeling multilevel structure for complex survey data is complicated because building a multilevel model is not an infallible statistical strategy unless the hypothesized model is close to the real data structure. Methodologists have suggested using different modeling techniques to investigate potential multilevel structure of survey data. Using iMCFA, researchers can visually set the between- and within-level factorial structure to fit MCFA, CFA and/or MAX MCFA models for complex survey data. iMCFA can then yield between- and within-level variance-covariance matrices, calculate intraclass correlations, perform the analyses and generate the outputs for respective models. The summary of the analytical outputs from LISREL is gathered and tabulated for further model comparison and interpretation. iMCFA also provides LISREL syntax of different models for researchers' future use. An empirical and a simulated multilevel dataset with complex and simple structures in the within or between level was used to illustrate the usability and the effectiveness of the iMCFA procedure on analyzing complex survey data. The analytic results of iMCFA using Muthen's limited information estimator were compared with those of Mplus using Full Information Maximum Likelihood regarding the effectiveness of different estimation methods.

## Introduction

Confirmatory Factor Analysis (CFA) has been widely utilized to examine the factorial structure of measures/scales in behavioral, sociological, educational, and organizational fields (Thompson, 2004; Kaplan, 2008; Kline, 2016). Researchers utilize CFAs to examine the reliability and validity of the underlying structure of test items and the theoretical constructs (Raykov, 2004; Raykov and Marcoulides, 2006; Geldhof et al., 2013). A fundamental assumption of the CFA analysis is that the responses from participants are independently and identically distributed (Bollen, 1989; Kaplan, 2008; Kline, 2016). However, the independence assumption can hardly be met for the survey dataset in the empirical studies. For instance, in educational and organizational research, we might utilize the complex survey sampling strategy (e.g., multistage sampling, cluster sampling, etc.) to collect the responses of an individual or lower sampling unit, which are nested within between-level clusters/groups (Stapleton, 2006, 2008; e.g., Wu et al., 2014; Wu, 2017). Within this context, participants in the same group with the same cluster information might yield more homogenous responses than those in different groups (Bovaird, 2007). Using the CFA without considering the dependent/multilevel structure in the complex survey data will result in biased parameter estimates and erroneous standard error estimates as well as inconsistent statistical inferences of the analytic results (Muthén and Satorra, 1995; Stapleton, 2008; Wu and Kwok, 2012; Wu et al., 2017).

In order to examine the multilevel factorial structure of complex survey data, various CFA techniques have been proposed, such as the model-based approach (Multilevel CFA, MCFA, e.g., Muthén, 1991; Hox, 1993; Mehta and Neale, 2005) and the maximum model in CFA (MAX CFA, e.g., Ryu and West, 2009; Wu and Kwok, 2012). The MCFA builds up a hierarchical statistical model corresponding to the multilevel structure of the complex survey data, so that the within-cluster and between-cluster model parameters can be separately and freely estimated (Muthén and Satorra, 1989). MAX MCFA is a special case of MCFA (Ryu and West, 2009; Wu and Kwok, 2012), which is usually considered as a partially saturated model during the process of building up a valid MCFA model (Hox, 2010). When using the maximum modeling strategy, researchers build up a MCFA model with a saturated between-level and a hypothesized within-level model. By doing so, all the unique elements of the variance-covariance matrix in the between-level will be estimated with the consumption of all the available degrees of freedom. Therefore, the saturated and just-identified between-level model contributes nothing to the fitting function (Hox, 2010), which allows us to diagnose the misspecification of the within-level model with the level-specific model-fit information (Ryu and West, 2009). These two approaches have been shown to yield consistent parameter estimates and statistical inferences as the population multilevel model (Wu and Kwok, 2012). However, modeling the multilevel structure of complex survey data is more complicated and requires more advanced statistical techniques and specific computer software.

The purpose of this study is threefold. First, the study intends to provide an integrated software for flexible multilevel modeling with Lisrel v.8 and below. Second, we investigate the performance of CFA, MCFA, and MAX MCFA in analyzing multilevel data with a complex within and simple between structure^1^ as well as a complex between and simple within structure. Third, we compare analysis results for the three modeling techniques using Muthen's limited information estimator (MUML in iMCFA) and Full Information Maximum Likelihood (FIML in Mplus). A review of literature on different ways of multilevel model construction and constraints of the current SEM software was provided, followed by the demonstration of iMCFA (i.e., the integrated Multilevel Confirmatory Analysis program).

### Multilevel model construction

Researchers have constructed MCFA models in two major approaches. For the first approach, they separated the level-varying covariance components from total covariance structures and used the level-specific covariance component to build the specific-level models (Muthén, 1994; Yuan and Bentler, 2007). For the second approach, they used the maximum model (or the unrestricted/saturated model) as the baseline to construct the between-level model with theoretical evidence (Yuan and Bentler, 2003; Stapleton, 2008; Hox, 2010).

The basic idea of MCFA is to decompose the total variance-covariance matrix, **Σ**_*T*_, into between-level variance-covariance (V-C) matrix, **Σ**_*B*_, and within-level V-C matrix, **Σ**_*W*_. Assuming **y**_gi_ is the observed variables for participant i within cluster g, the total V-C matrix **Σ**_*T*_ = *Var*[**y**_gi_]. The corresponding between- and within-level V-C components will be orthogonal and additive (Searle et al., 1992; Muthén, 1994). Same score decomposition can be performed for the observed complex survey sample data, and the resulted sample V-C matrix can be shown as,

$$S_{T}=S_{B}+S_{W}$$

where **S**_**B**_ and **S**_**W**_ are the level-varying V-C estimators to their population counterparts, **Σ**_**B**_ and **Σ**_**W**_, respectively (Muthén, 1994; Hox, 2002; Hox and Maas, 2004; Heck and Thomas, 2008). With the variance-covariance matrix decomposition, Muthén (1989, 1990) presented an a partial Maximum likelihood estimation method, also named MUML (Muthén's limited information estimator). In MUML, two variance-covariance matrices of different levels are constructed as

(1) $$S_{T}=S_{B,MUML}+S_{PW,MUML}$$

Consider a multilevel dataset with the sample size of *N*, i.e., on average *N*_*g*_ participants nested within respective *G* groups. The above three variance-covariance matrices are defined as

(2) $$\begin{array}{l} S_{T}=\frac{1}{N-1}\sum_{g\;=\;1}^{G}\sum_{i\;=\;1}^{N_{g}}\left(y_{gi}-\bar{y}\right){\left(y_{gi}-\bar{y}\right)}^{\prime }\\ S_{PW,MUML}=\frac{1}{N-G}\sum_{g\;=\;1}^{G}\sum_{i\;=\;1}^{N_{g}}\left(y_{gi}-{\bar{y}}_{g}\right){\left(y_{gi}-{\bar{y}}_{g}\right)}^{\prime }\\ S_{B,MUML}=\frac{1}{G-1}\sum_{g\;=\;1}^{G}\left({\bar{y}}_{g}-\bar{y}\right){\left({\bar{y}}_{g}-\bar{y}\right)}^{\prime } \end{array}$$

where the grand mean $\bar{y}=\frac{1}{N}\sum_{g\;=\;1}^{G}\sum_{i\;=\;1}^{N_{g}}y_{gi}$ and group mean of *g*^th^ group ${\bar{y}}_{g}=\frac{1}{N_{g}}\sum_{i\;=\;1}^{N_{g}}y_{gi}$.

In Equation (2), Muthén showed that the pooled within-level observed variance-covariance matrix **S**_**PW,MUML**_ is the consistent and unbiased estimator to **Σ**_**W**_, and the scaled between-level observed variance-covariance matrix **S**_**B**,**MUML**_ is the consistent and unbiased estimator to **Σ**_**W**_ + *c***Σ**_**B**_, where $c={\left(N\left(G-1\right)\right)}_{}^{-1}\left(N^{2}-\overset{G}{\underset{g}{\sum }}n_{g}^{2}\right)$ is close to the averaged group size. In a balance design (i.e., all between-level units have the same group size), MUML is the same as the original unbiased ML estimator. But in an unbalance design, MUML is the simplified version of quasi-ML estimation method (Varin and Vidoni, 2005) and only uses a common group size, *c*, as the weighting scalar of the between-level variance component in the likelihood function, that is,

(3) $$\begin{array}{l} F_{MUML}\left(\Sigma ,\hat{\Sigma }\right)=F_{MUML}\left(S,\hat{\Sigma }\right)=G\{\ln\left|{\hat{\Sigma }}_{W}+c{\hat{\Sigma }}_{B}\right|\\ \;+\;tr\left({\left({\hat{\Sigma }}_{W}+c{\hat{\Sigma }}_{B}\right)}^{-1}S_{B}\right)-\ln\left|S_{B}\right|-p\}\\ \;+\;\left(N-G\right)\{\ln\left|{\hat{\Sigma }}_{W}\right|+tr\left({\hat{\Sigma }}_{W}^{-1}S_{PW}\right)\\ \;-\;\ln\left|S_{PW}\right|-p\} \end{array}$$

MUML is also called as limited information or quasi-maximum likelihood estimation because it assumes that all groups have equal group size, even though they may not. Researchers can use the MUML in Mplus (Muthén and Muthén, 1998-2017) with the routine of “ESTIMATOR = MUML.”

Due to the limitation of conducting MCFA analyses in LISREL v.8 and below, we can decompose the between- and within-level variance-covariance structures shown in Equation (2). One nice feature about MUML is that researchers can use the multi-group analysis routine provided in various SEM programs to conduct the multilevel CFA analysis. Researchers need to separate the original data into two groups: the between-level group with between-level V-C matrix **S**_**B, MUML**_ and group number *G*, and the within-level group with within-level V-C matrix **S**_**PW, MUML**_ with sample size *N-G*. The multilevel data can then be analyzed with the multi-group routine. The detailed steps of this process is provided in Heck and Thomas (2008) and Muthén (1994). Compared with Full-information Maximum Likelihood estimator (FIML, Arbuckle, 1996; Mehta and Neale, 2005), MUML is simpler in computing the parameter estimates while FIML is computationally heavier as the size of sub-groups increases. Muthén and Satorra (1995) concluded that MUML generally performs equally well as FIML in various conditions; however, Hox and Maas (2004) showed FIML has more accurate parameter estimates than MUML does. We will check the analytical result consistency between iMCFA using MUML and Mplus using FIML with unbalanced- and balanced-design^2^ samples in the provided scenarios.

### Multilevel SEM modeling software

With the advance of software packages, researchers now are more comfortable to build up multilevel models in their research practice (Hox, 2010; for comprehensive review of available software and packages, please refer to Goldstein, 2010; Snijders and Bosker, 2011). For example, an newly developed R package, xxM (Mehta, 2013), can be used to estimate multilevel SEM models featuring complex level-dependent data structures. The xxM is based on OpenMx (Boker et al., 2017) and a framework called n-Level Structural Equation Modeling (NL-SEM, e.g., Ryu and Mehta, 2017) which allows specifying multilevel models with observed and latent variables. Mplus (Muthén and Muthén, 1998-2017) and LISREL (Jöreskog et al., 2001) are commonly used structural equation modeling software for MCFAs. Researchers can use those software to examine the level-varying factorial structures, and simultaneously test different-level hypotheses (Muthén, 1994) with distinct model specifications. These programs present the overall model fit test statistics and fit indices with the provided multilevel SEM routines (e.g., TYPE = TWOLEVEL in Mplus), which cannot reveal possible misfit in respective levels. Instead, researchers can use partially saturated model (e.g., MAX MCFA in this study) or adjust the multi-group comparison approach (Muthén, 1994; Yuan and Bentler, 2007) to obtain level-specific model fit indices and test statistics in any SEM software. However, the programming of multilevel modeling practice would be intimidating to some researchers. Moreover, researchers can only specify the MCFA model with the same between- and within-level structure with Lisrel v.8 and below using SIMPLIS syntax via multi-group comparison (Jöreskog and Sörbom, 2004). To perform a MCFA with different factorial structures in the between and within level, researchers had to apply the LISREL syntax with matrix specification. Although the SURVEYGLIM procedure can be used to obtain the between-level and within-level covariance matrices after LISREL v.8.3 (Jöreskog et al., 2001), constructing MCFA using LISREL is still a daunting task which requires statistical computing operation in a multi-group comparison setting and LISREL coding in a matrix form.

Therefore, with the above-mentioned issues, researchers are in need of an effective and flexible multilevel modeling software which allows result comparison among competing models for optimal model selection.

### Modeling multilevel CFA models using iMCFA

Methodologists have provided suggestions and guidelines for constructing multilevel SEM models. Muthén (1994) proposed a stepwise procedure for multilevel model construction. He suggested that, in lack of model fit test and indices result for conventional CFA model, researchers should compute the intraclass correlation (ICC) measures for complex survey data. If the ICC value is nonzero or larger than certain thresholds (Muthén, 1994; Hedges and Hedberg, 2007; Hox, 2010), researchers should then build the multilevel model with respective within- and between-level structures. Hox (2002) suggested to compare the overall model-fit χ^2^ test statistics of the one-level CFA (i.e., the null model) and of the independent MCFA (i.e., the MCFA with only variance estimates of between-level indicators and a hypothesized within-level model) with the saturated MCFA (i.e., MAX MCFA) as the first step to decide whether researchers should move on to establish a MCFA. Still other researchers (Yuan and Bentler, 2007; Ryu and West, 2009) provided level-specific model fit test statistics and fit indices to detect possible between level variation and potential model miss-specifications at respective levels.

The above-mentioned studies involved the design of the balanced synthetic dataset under the segregating approach (Yuan and Bentler, 2007) or the partially saturated model approach (Ryu and West, 2009) to capitalize on the importance of building adequate models with respective to the different level structures in analyzing the complex survey data. Constructing multilevel modeling according to the complex sampling design of the survey data could prevent erroneous inferences on the parameter estimates (Muthén, 1994; Yuan and Bentler, 2007), especially under a scenario with level-varying structures, in which the factor structure at the between level is different from that at the within level (Wu and Kwok, 2012). Besides, the precision of the inference of the relationship between items and factors, the scale reliability of constructs, and the variance explained of items would also be secured if we specify adequate multilevel models on the survey data (e.g., Raykov, 2004; Raykov and Marcoulides, 2006; Geldhof et al., 2013). Thus, we provide iMCFA using Lisrel v8.8 or below to help researchers build up valid multilevel models and to obtain ICC, variance-covariance matrix for separate level, and tabulated model comparison results on different modeling strategies^3^.

In this study, we provide a general three-step procedure to construct a valid MCFA for complex survey data with level-varying structures and included the comparison of fit statistics and indices among three competing models for more precise model construction using iMCFA. At the first step, researchers should evaluate the model fit information of CFA as well as the congruency of the within-level parameter estimates between CFA and MAX MCFA. If CFA demonstrates bad overall model fit information or produces incongruent parameter estimates (especially the random effect estimates, e.g., factor variance or indicator residual variance) to the within-level of MAX MCFA, this is a strong message of potential between-level variation and level-varying structures. Next, researchers should focus on specifying within-level model using MAX MCFA by referencing to the model-fitχ^2^ statistic and fit indices. After the within-level model is set, researchers can then proceed to construct a valid between-level model using MCFA based on the between-level model fit information at the third step with theoretical and empirical supports. An integrated MCFA program (iMCFA) is provided to manage the dataset and to perform the one-level CFA, MAX MCFA, and MCFA with Lisrel v.8 and below, which can aid the process of model selection and comparison. Two unbalanced and balanced datasets with level-varying structures (Study 1: the empirical unbalanced family IQ dataset with simple between and complex within structure; Study 2: the simulated balanced dataset with complex between and simple within structure) were used to illustrate the effectiveness and efficiency of the proposed approach and tool in building a valid MCFA model.

## Methods

We developed iMCFA to perform CFAs for complex survey data. The program is written in c++/CLI (Common language interface) in Microsoft Visual Studio 2012 (Microsoft Co. Ltd.). Researchers can use iMCFA to set the between- and within-level CFA models according to the theories and experiences, and to perform MCFA, CFA and/or MAX MCFA. Besides the computation of the level-varying variance-covariance matrices and automatic generation and execution of LISREL syntax, iMCFA will tabulate the results among the three competing models for further statistical decision making.

### Performing CFA analyses using iMCFA

**Figure 1:**
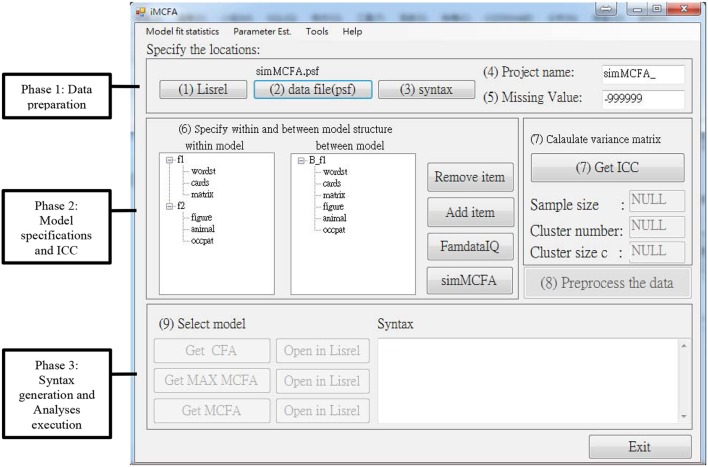
Snapshot of iMCFA interface with an example of two-level CFA model with one factors at between level and two factors at within level.

The snapshot of user interface of iMCFA is shown in Figure 1.

The interface of iMCFA is divided into three main phases, Phase 1: Data preparation, Phase 2: Model specifications and ICC calculation, and Phase 3: Syntax generation and Analyses execution. In the first phase, users need to specify the folder of LISREL program in Step (1), which contains LISREL^*^.exe, LisWin32.exe, and multilev^*^.exe, the folder of dataset file in Step (2) and the folder to save the generated syntax in Step (3). The default input data format for iMCFA is the LISREL data format (^*^.psf). Besides study variables, the imported dataset must include a Cluster ID variable and a Case ID variable, which identify the between-level and within-level sample units. The dataset should be sorted ascendingly according to Cluster ID and Case ID. iMCFA provides a tool to generate the Case ID variable. This tool can also convert the dataset from pure text format (^*^.dat) into LISREL data format (^*^.psf). The project name in Step (4) will be used as the prefix in the file name of all generated files, including the files of syntax, output, and variance-covariance matrix of variables. In Step (5), users have to specify a value of missing data (the default value will be−999999) to complete the data preparation phase.

In Phase 2 of model specification, Step (6) required researchers to specify the between- and within-level CFA structures. Users can add or remove latent and manifest variables after the data are read in. The variable labels can be edited and should be less than seven characters. After completing the above steps, click the ‘Get ICC’ button at Step (7) to do the ICC analysis and save information of sample size, cluster number, cluster size c, and variance-covariance matrix **S**_**PW**_, **S**_**B**_, **S**_**B,MUML**_ and **S**_**T**_ for the following analyses. At Step (8), the iMCFA gathers and saves all the matrices and values into the database for the following MAX MCFA, CFA, and MCFA analyses. For experienced researchers, these matrices can be used to conduct the multilevel analysis using any analytical programs with the distinct level-varying covariance matrices.

**Figure 2:**
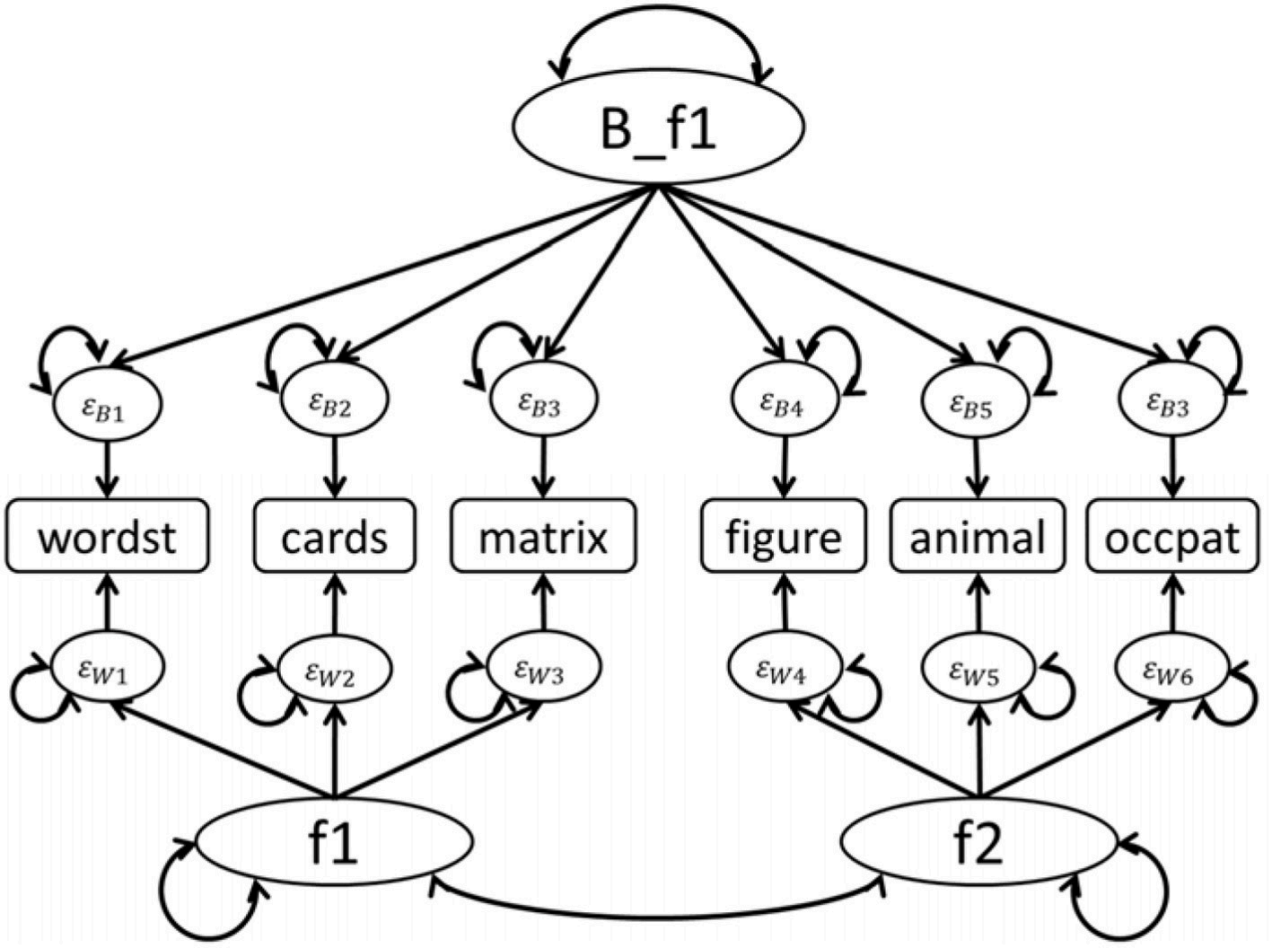
MCFA model with 1 factor at between level and 2 factors at within level for FamdataIQ dataset.

At Step (9) of Analysis and Syntax Phase 3, researchers can execute MAX MCFA, CFA, and MCFA separately. For MAX MCFA models, the within-level structure is based on the specification of the within-level model at Step (6), and the between-level structure is saturated, meaning all the between-level indicators are inter-correlated. For CFA models, the one-level structure is based on the specification of within-level model at Step (6). Researchers can specify MCFA model with unequal between- and within-level structures using iMCFA. For example, we specify a MCFA model (shown in Figure 2) with one between-level factor and two within-level factors. To correctly perform the MCFA, researchers need to keep the order and the number of items the same in between- and within-level model specified at step (6).

LISREL syntax for three models will be generated and executed after clicking the buttons of “Get MAX MCFA,” “Get CFA,” and “Get MCFA.” The model goodness-of-fit test statistics and indices and parameter estimates will be retrieved from the output at this step. The syntax will be presented in the bottom-right text box. For convenience, users can click the “Open in LISREL” button to execute the corresponding syntax in LISREL, which can generate the analytic result and the model diagram.

On the top of the panel, researchers can request the tabulated analysis results by clicking tabs of “Model fit statistics” and “Parameter Est.” The Model fit statistics tab shows the fit test statistic and fit index information for three models. The Parameter Est. tab summarizes the estimates of factor loadings, residual variances of each item, and the covariances among latent factors for three models. Researchers are allowed to save these tables in a text file, which can be found in the same folder of syntax files.

In the following sections, we used two datasets to demonstrate iMCFA, one was a family IQ dataset (famdataIQ.psf, Hox, 1993, 2010) and the other was a simulated dataset (simMCFA.psf, Wu and Kwok, 2012). The commonly-used criteria of model fit indices were used to assess the goodness of fit of the proposed models to the dataset.

### Study 1: empirical unbalanced dataset with simple between and complex within structure

#### Data description

The empirical dataset (famdataIQ.psf) is from the dissertation study of Van Peet (1992) which also appeared in Hox (1993, 2010). Data were collected on 400 children nested within 60 families with a minimum 4 and maximum 12 children in each family (*M* = 6.67). The instrument used was the Groninger Intelligent Test (GIT) which consisted of six subtests, including wordlist, laying cards, matrices, hidden figures, naming animals, and naming occupations. Strong correlation among members in a family were expected because intelligence is assumed to be greatly influenced by heredity and environments. Scores on the six subtests for this hierarchical data were then divided into family level and individual level variables. According to Hox (1993), there was a common factor in the family level due to shared genetic and environmental influences while in the individual level two separate factors existed to explain the idiosyncrasy in each individual's intelligence.

#### Model specification

The famdataIQ.psf involved eight variables: family id, user id, wordlist (wordst), laying cards (cards), matrices (matrix), hidden figures (figure), naming animals (animal), and naming occupations (occpat). We constructed two factors (f1 and f2) in the within level and one factor (B_f1) in the between level based on Hox (1993). In the within level, wordst, cards, and matrix were loaded on f1, while figure, animal, and occpat were loaded on f2. In the between level, all six items were loaded on B_f1 as shown in Figure 2.

#### Result for study 1

**Figure 3:**
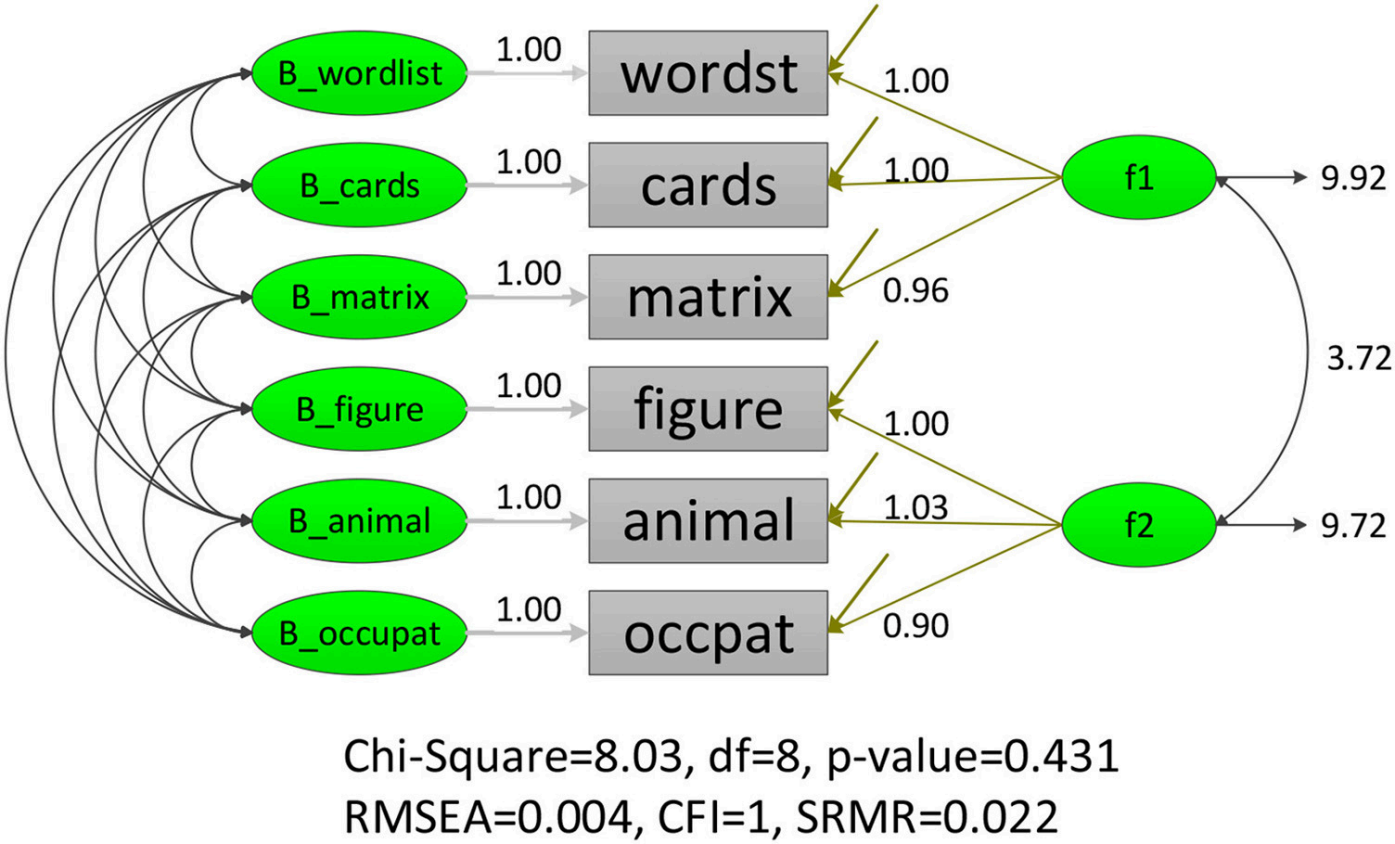
LISREL illustration of MAX MCFA model on FamdataIQ dataset.

Three modeling techniques in iMCFA were applied to analyze the family IQ dataset. All models had adequate model-fit test statistic and fit indices for the data with unequal family- and individual-level structure (e.g., for MCFA, χ^2^ = 7.920 with *df* = 9, CFI = 1.000, RMSEA < 0.001, SRMR = 0.012; for MAX MCFA, χ^2^ = 8.027 with *df* = 8, CFI = 1.000, RMSEA = 0.004, SRMR = 0.022; for CFA, χ^2^ = 10.241 with *df* = 8, CFI = 0.999, RMSEA = 0.027, SRMR = 0.016;). The ICCs for six indicators were larger than 0.369 (as shown in Table 1), which indicated potential between-level variation and the need to use multilevel CFA techniques (Hox, 2010). The path diagrams with analytical result of MCFA and MAX CFA are illustrated in Figure 3, 4. The results of MCFA confirmed the existence of a general between-level intelligence construct, which could explain the influence of heredity and environment in a family. The three modeling techniques exhibited a 2-factor structure in the within-level model representing the idiosyncrasies in each individual's intelligence (Van Peet, 1992; Zimprich and Martin, 2009). We then compared the performance of these three modeling techniques on this complex survey dataset.

**Table 1:** ICC and *R*^2^-values of indicators of three models for FamdataIQ dataset (*N* = 400, *G* = 60).

	**Wordst**	**Cards**	**Matrix**	**Figure**	**Animal**	**Occpat**
ICC	0.399	0.408	0.369	0.374	0.419	0.503
*R*^2^_MCFA						
Individual-level	0.614	0.651	0.589	0.588	0.678	0.596
Family-level	0.885	0.874	0.781	0.787	0.937	0.880
*R*^2^_ CFA	0.735	0.733	0.657	0.649	0.789	0.737
*R*^2^_MAX CFA	0.614	0.651	0.589	0.588	0.678	0.596

**Figure 4:**
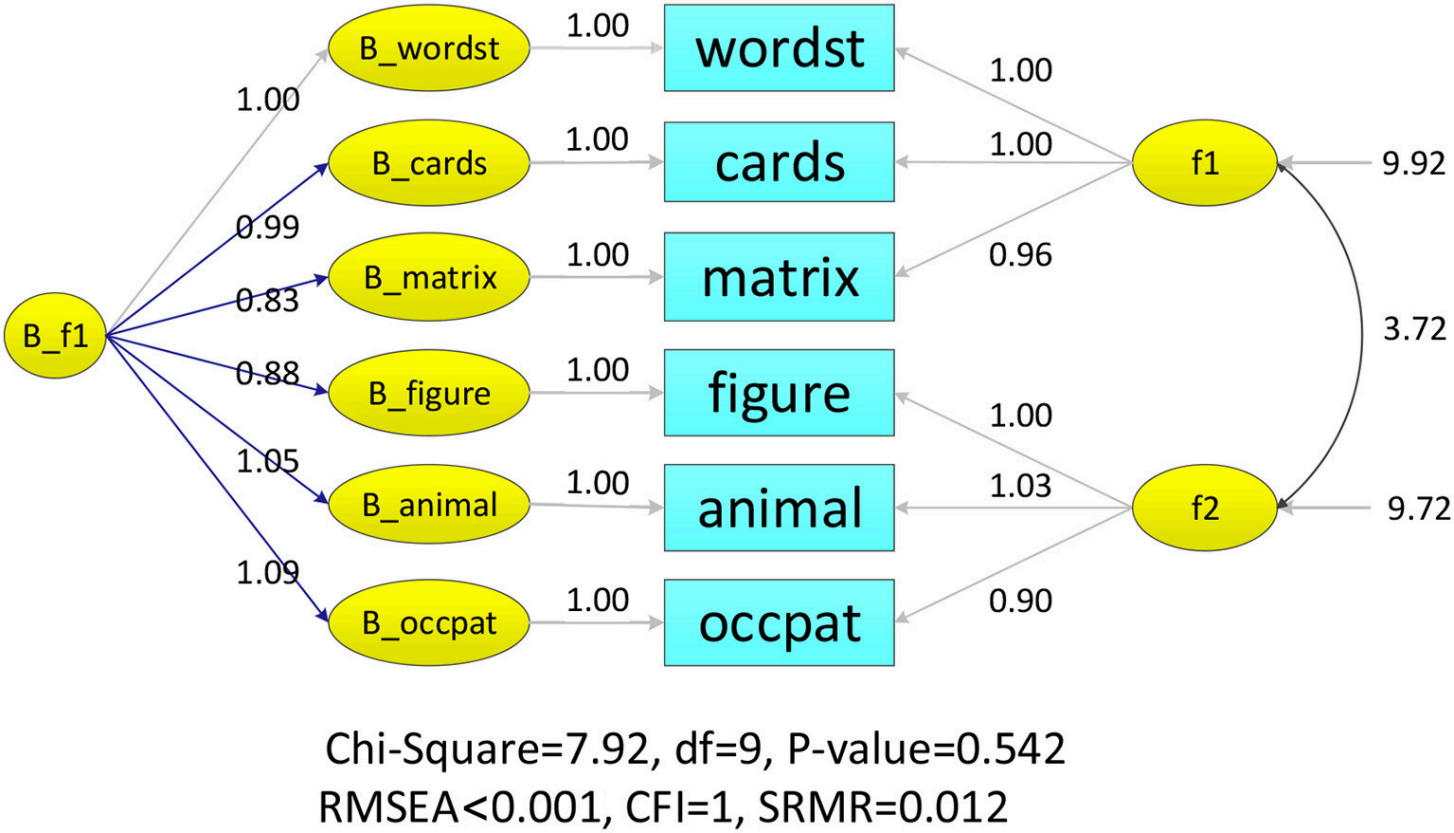
LISREL illustration of MCFA model on famdataIQ dataset.

The analysis result of three modeling techniques was tabulated in Table 2. The MAX MCFA model yielded similar model evaluation result and parameter estimates as the MCFA in the within level. However, when CFA was applied on this family dataset, the factor loading estimates were statistically different from those of the MAX MCFA model or MCFA [e.g., ${\hat{\lambda }}_{occupats,W\text{\_}IQ2}^{MCFA}={\hat{\lambda }}_{occupats,W\text{\_}IQ2}^{MAX}=0.901$, vs. ${\hat{\lambda }}_{occupats,W\text{\_}IQ2}^{CFA}=1.071$, *t*_(*df*)_ = 1.99(798), *p* = 0.046]. The relative difference of factor loading estimates for CFA compared to the MAX MCFA and MCFA ranged from −6.18 to 15.87%, which could be considered as a moderate to substantial difference (Flora and Curran, 2004). This level of incongruence between parameter estimates of CFA and MAX MCFA might indicate the necessity of constructing a multilevel model with level-varying structures for this dataset.

**Table 2:** Three CFA models of empirical famdataIQ dataset. (*N* = 400, *G* = 60).

	**MCFA**		**CFA**		**MAX MCFA**	
Chi-square (df)	7.920(9)		10.241(8)		8.027(8)	
CFI	1.000		0.999		1.000	
RMSEA	0.000		0.027		0.004	
SRMR	0.012		0.016		0.022	
	**Est**.	**SE**	**Est**.	**SE**	**Est**.	**SE**
**INDIVIDUAL LEVEL**						
**W_IQ1 by**						
wordlist	1		1		1	
cards	1.001^***^	0.069	0.979^***^	0.049	1.001^***^	0.069
matrices	0.962^***^	0.068	0.906^***^	0.048	0.962^***^	0.068
**W_IQ2 by**						
figures	1		1	0	1	0
animals	1.026^***^	0.071	1.093^***^	0.056	1.026^***^	0.071
occupats	0.901^***^	0.064	1.071^***^	0.056	0.901^***^	0.064
*Cov(W_IQ1,W_IQ2)*	3.721^***^	0.658	12.622^***^	1.344	3.721^***^	0.658
*Var(W_IQ1)*	9.918^***^	1.173	19.755^***^	1.937	9.918^***^	1.173
*Var(W_IQ2)*	9.724^***^	1.179	17.136^***^	1.828	9.724^***^	1.179
***RESIDUAL VAR***						
*wordlist*	6.228^***^	0.677	7.131^***^	0.799	6.228^***^	0.677
*cards*	5.335^***^	0.637	6.881^***^	0.768	5.355^***^	0.637
*matrices*	6.414^***^	0.659	8.483^***^	0.800	6.414^***^	0.659
*figures*	6.824^***^	0.696	9.286^***^	0.840	6.824^***^	0.696
*animals*	4.859^***^	0.625	5.489^***^	0.703	4.859^***^	0.625
*occupats*	5.358^***^	0.556	7.016^***^	0.757	5.358^***^	0.556
**FAMILY LEVEL**						
**B_IQ by**						
wordlist	1					
cards	0.985^***^	0.083				
matrices	0.831^***^	0.080				
figures	0.878^***^	0.109				
animals	1.050^***^	0.113				
occupats	1.091^***^	0.118				
*Var(B_IQ)*	9.677^***^	1.797				
***RESIDUAL VAR***						
*wordlist*	1.024ns	0.760				
*cards*	1.449^*^	0.728				
*matrices*	1.947^*^	0.767				
*figures*	2.161^**^	0.813				
*animals*	0.495 ns	0.662				
*occupats*	1.763^*^	0.759				

* *p < 0.05*,

** *p < 0.01*,

*** *p < 0.001*.

*χ^2^, Chi-square value; df, Degrees of freedom; CFI, Comparative fit index; RMSEA, Root mean square error of approximation; SRMR, Standardized root mean square residual. The normal font denotes the fixed effect and intercept estimate; the italic denotes the random effect estimate*.

As for the random effect estimate, CFA generated an overall estimate of factor variance, which was roughly the summation of the family- and individual-level variance components (e.g., ${\text{Ψ}}_{wordst}^{CFA}\;=\;7.131$ equals ${\text{Ψ}}_{wordst,W}^{MCFA}\;=\;6.228$ plus ${\text{Ψ}}_{wordst,B}^{MCFA}\;=\;1.024$) and the MAX MCFA yielded consistent individual-level factor variance to the MCFA (e.g., ${\text{Ψ}}_{wordst,W}^{MCFA}={\text{Ψ}}_{wordst,W}^{MAX}=6.228$). However, CFA tended to generate inflated *R*^2^ for the within-level indicators compared to MCFA and MAX MCFA.

We also compared the parameter estimates of three proposed CFA modeling techniques using iMCFA with MUML variance decomposition and those obtained from Mplus 6.11 with FIML estimation (as shown in Table S.1 in the Appendix). For the parameter estimates in the within-level model, the averaged relative bias was 0.022% (SD = 0.543%) for MCFA, 0.040% (SD = 0.126%) for MAX MCFA, and 1.037% (*SD* = 1.827%) for CFA. The relative bias of estimates between these two programs for multilevel CFAs could be deemed as trivial (Flora and Curran, 2004).

### Study 2: simulated balanced dataset with complex between and simple within structure

#### Data description

The simMCFA.psf involved nine indicators (V1 to V9). In the population model, all nine indicators were loaded on one factor (W_f1) at the within level and three factors (B_f1, B_f2, and B_f3) at the between level. This simulated balanced dataset was generated using Monte Carlo procedure of Mplus 6.11 with 10,000 observations nested within 50 groups (i.e., each group had 200 participants). All factor loadings were set at 0.80, and the residual variances of outcome variables were fixed at 0.36 in both within- and between-level models. Moreover, covariances among three between-level latent factors were fixed at 0.30. The ICCs in Table 3 for nine indicators were larger than 0.388. The detailed settings of the true model with cross-loaded factor loadings could be referred to scenario 3 in Wu and Kwok (2012).

**Table 3:** ICC and *R*^2^-values of nine indicators of three models for simMCFA dataset.

	**V1**	**V2**	**V3**	**V4**	**V5**	**V6**	**V7**	**V8**	**V9**
ICC	0.516	0.516	0.535	0.487	0.388	0.484	0.353	0.470	0.456
*R*^2^_MCFA									
Within-level	0.642	0.641	0.636	0.637	0.640	0.638	0.633	0.635	0.639
Between-level	0.737	0.675	0.783	0.639	0.675	0.420	0.662	0.593	0.818
*R*^2^_ CFA	0.616	0.577	0.601	0.468	0.439	0.372	0.424	0.421	0.434
*R*^2^_ MAX_MCFA	0.642	0.641	0.636	0.637	0.640	0.638	0.633	0.635	0.639

#### Result for study 2

The same three modeling techniques with a simple structure in the within level were applied for the simulated dataset. The analysis results were tabulated in Table 4. Furthermore, we also constructed a misspecified MCFA with one factor in both between- and within-level model, that is, the between-level model did not confirm to the true three-factor structure. Likewise, the correctly-specified MCFA and MAX MCFA models yielded similar model evaluation results and parameter estimates; however, CFA yielded inadequate overall model-fit test statistic and fit indices (For CFA, overall χ^2^ = 12699.87 with *df* = 27, CFI = 0.881, RMSEA = 0.217 SRMR = 0.090; For MAX MCFA, within-level χ^2^ = 26.089 with *df* = 27, CFI = 1.000, RMSEA < 0.001, SRMR = 0.003; For MCFA, the between-level χ^2^ = 824.5 with *df* = 24, CFI = 0.991, RMSEA = 0.058, SRMR = 0.027). When the CFA was applied, the factor loading estimates deviated from those of the MCFA and the MAX MCFA [e.g., ${\hat{\lambda }}_{V5,f1}^{MCFA}=0.802$ and ${\hat{\lambda }}_{V5,f1}^{MAX}=0.802$ vs. ${\hat{\lambda }}_{V2,f1}^{CFA}=0.603$, *t*_(*df*)_ = 15.63(19998), *p* < 0.001], and the relative bias of factor loading estimates for the CFA comparing to the MCFA and MAX MCFA ranged from −38.47 to 1.20%, which could be seen as trivial to substantial differences (Flora and Curran, 2004). Both the model lack-of-fit information and the incongruence of parameter estimates inform the need of further multilevel modeling with level-varying structures. Researchers can use maximum modeling techniques with the within-level model goodness-of-fit tests and indices to construct a valid within-level model, and proceed to use MCFA with the respective between-level model-fit information to have a valid between-level model.

**Table 4:** Fit information and parameter estimates of hypothesized and misspecified models on dataset.

		**MCFA**		**MISS MCFA**		**CFA**		**MAX MCFA**	
Chi-square (df)		824.499(24)		7897.358(27)		12699.87(27)		26.089(27)	
CFI		0.991		0.931		0.881		1.000	
RMSEA		0.058		0.171		0.217		0.000	
SRMR		0.027		0.204		0.090		0.003	
		**Est**.	**SE**	**Est**.	**SE**	**Est**.	**SE**	**Est**.	**SE**
**WITHIN LEVEL**									
W_f1 by	V1	0.800	—	0.800	—	0.800	—	0.800	—
	V2	0.799	0.009	0.799	0.009	0.774	0.010	0.799	0.009
	V3	0.792	0.009	0.792	0.009	0.801	0.010	0.792	0.009
	V4	0.791	0.009	0.791	0.009	0.673	0.010	0.791	0.009
	V5	0.802	0.009	0.802	0.009	0.603	0.009	0.802	0.009
	V6	0.794	0.009	0.794	0.009	0.599	0.010	0.794	0.009
	V7	0.798	0.009	0.798	0.009	0.576	0.009	0.798	0.009
	V8	0.787	0.009	0.787	0.009	0.625	0.009	0.787	0.009
	V9	0.798	0.009	0.798	0.009	0.633	0.009	0.798	0.009
*Var(W_f1)*		1.001	0.021	1.001	0.021	1.985	0.044	1.001	0.021
***RESIDUAL VAR***									
	*V1*	0.357	0.006	0.357	0.006	0.791	0.014	0.357	0.006
	*V2*	0.358	0.006	0.358	0.006	0.872	0.015	0.358	0.006
	*V3*	0.359	0.006	0.359	0.006	0.848	0.014	0.359	0.006
	*V4*	0.358	0.006	0.358	0.006	1.020	0.016	0.358	0.006
	*V5*	0.362	0.006	0.362	0.006	0.920	0.014	0.362	0.006
	*V6*	0.358	0.006	0.358	0.006	1.205	0.018	0.358	0.006
	*V7*	0.369	0.006	0.369	0.006	0.895	0.014	0.369	0.006
	*V8*	0.356	0.006	0.356	0.006	1.066	0.016	0.356	0.006
	*V9*	0.360	0.006	0.360	0.006	1.037	0.016	0.360	0.006
**BETWEEN LEVEL**									
B_f1 by	V1	0.800	—	0.800	—				
	V2	0.802	0.015	1.191	0.099				
	V3	0.879	0.017	1.075	0.092				
B_f2 by	V4	0.800	—	2.196	0.174				
	V5	0.601	0.019	1.557	0.122				
	V6	0.566	0.019	1.592	0.128				
B_f3 by	V7	0.800	—	1.894	0.199				
	V8	0.973	0.025	2.198	0.224				
	V9	1.139	0.031	2.496	0.250				
*Var(B_f1)*		1.253	0.045	0.051	0.009				
*Cov(B_f1,B_f2)*		0.552	0.031						
*Cov(B_f1,B_f3)*		1.025	0.048						
*Var(B_f2)*		0.305	0.022						
*Cov(B_f2,B_f3)*		0.036	0.021						
*Var(B_f3)*		0.538	0.029						
*Residual Var*									
	*V1*	0.292	0.014	0.593	0.015				
	*V2*	0.347	0.014	0.620	0.016				
	*V3*	0.240	0.015	0.634	0.016				
	*V4*	0.270	0.018	0.400	0.015				
	*V5*	0.238	0.012	0.379	0.013				
	*V6*	0.569	0.016	0.672	0.017				
	*V7*	0.193	0.011	0.195	0.011				
	*V8*	0.343	0.014	0.338	0.014				
	*V9*	0.149	0.016	0.199	0.014				

*(N of sample = 10,000 with Group Number = 50 and Group Size = 200)*.

*All above parameter estimates are statistically significant at the level of p < 0.05*.

*χ^2^, Chi-square value; df, Degrees of freedom; CFI, Comparative fit index; RMSEA, Root mean square error of approximation; SRMR, Standardized root mean square residual. The normal font denotes the fixed effect and intercept estimate; the italic denotes the random effect estimate. MCFA: the multilevel CFA model with one within-level factor and three between-level factors as the population model of simulation dataset. MISS MCFA: the miss-specified multilevel CFA model with one within-level factor and one between-level factor. CFA: the miss-specified CFA model with a uni-factor single-level structure. MAX MCFA: the multilevel CFA model with one within-level factor and saturated between-level structure*.

Comparing the correctly- and miss-specified MCFAs, the model-fit χ^2^ statistic indicated that the misspecified MCFA model did not fit the data exactly, and the fit indices exhibited more severe model badness-of-fit result (For misspecified MCFA on the left hand side of Table 4, CFI = 0.931, RMSEA = 0.171, and SRMR = 0.204). With the valid within-level structure at the MAX MCFA step, researchers can then build up several competing MCFAs with different between-level models and conduct the model comparison analyses with the aid of model-fit χ^2^ and fit indices provided in the MCFA step of iMCFA to select the proper multilevel model with statistical and theoretical evidence.

CFA tended to generate smaller *R*^2^ for the within-level indicators compared to the MCFA and MAX MCFA models as shown in Table 3. We compared the parameter estimates of iMCFA with MUML and those of Mplus 6.11 with FIML (as shown in Table S.2 in the Appendix). For the individual-level model, the relative bias of MCFA, MAX MCFA, and CFA was very close to zero. Indicating the parameters estimates generated by the MUML were consistent with those generated by the FIML estimator.

## Discussion and conclusion

In order to reduce the complexity of using multilevel CFA techniques, we provided the iMCFA program as an integrated tool to manage three most commonly-used CFA modeling techniques, namely regular CFA, MAX MCFA, and MCFA, on a user-friendly interface to analyze complex survey data with LISREL v.8 and below. The capacity to specify level-varying structures is fundamental to ensure the accuracy of analytical results in various CFA analyses with complex survey dataset. Failing to build up a model conforming to the true multilevel data structure may lead to erroneous analytical results and incorrect conclusions (Wu and Kwok, 2012). Even with the advance of the analytical software on analyzing various SEM models, it is difficult for researchers to specify MCFA models with level-varying structures with the supports of model-fit test and fit indices. Specifically, there is still not an efficient function in these programs to compare the adequacy of different modeling techniques simultaneously on the multilevel data. In this study, we used the iMCFA program to compare the performance of MCFA, miss-specified MCFA, MAX MCFA and CFA on two different datasets (one empirical unbalanced and one simulated balanced dataset) considering their level-varying structure and balanced/unbalanced design.

The different analytical results of CFA compared with the MAX MCFA technique may indicate a potential between-level structure in the dataset. In the illustrations, we demonstrated that when the relative bias of within-level factor loading estimates of CFA and MAX MCFA was moderate to substantial (Flora and Curran, 2004), there could be level-varying structures in the complex survey data. For the multilevel dataset with level-varying structures, CFA generated conflated parameter estimates of fixed and random effects with overall variance-covariance matrix along with the inconsistent standard error estimates. Besides, due to the conflated estimates in the one-level modeling, the variance explained measures (e.g., *R*^2^) of CFA were different from the outputs of MCFA and MAX MCFA (Wu and Kwok, 2012; Geldhof et al., 2013; Wu et al., 2017). For a complex survey dataset, the association between the *R*^2^ generated by regular CFA and the *R*^2^ measures in respective between- and within-levels by MCFA models warrants future simulation and/or mathematical investigations.

With level-specific variance components, MCFA could only generate consistent results when the analytical model is close to the true multilevel structures in both between- and within-level models simultaneously; while with a saturated between-level model, MAX MCFA model could be utilized to construct the individual model consistent with the within-level structure of the true multilevel model. If researchers and practitioners fail to use modeling techniques that are congruent with the multilevel structure of the complex survey data, they should exert caution in interpreting or making inferences from a regular CFA and MCFA. Instead, researchers would benefit from the use of MAX MCFA model offered in iMCFA in dealing with the complex survey data. If researchers are interested in only the research question about the within-level model, they should use the result from MAX MCFA analysis to draw a conclusion for the variation of within-level sampling units.

If researchers aim to answer research questions concerning different levels of the dataset, they could start with a MAX MCFA model to build an optimal within-level model. Next, they could go further to specify their between-level structure using MCFA to capture the between-level variation in their complex survey dataset (Hox, 2002). The model-fit information are indicators of the quality of hypothesized between-level model.

To complete the above-mentioned steps for building up an adequate multilevel CFA model, researchers or practitioners can use iMCFA to conduct multilevel CFA with equal or unequal between- and within-level structures in an effective and efficient way. They can use the tabulated analytical results provided by iMCFA to compare the performance of the three modeling techniques and to select the optimal model for statistical inference. Researchers can further use the generated LISREL syntax to request model diagrams and perform more detailed and advanced analyses in LISREL v.8 and below. The generated LISREL syntax of the MAX MCFA model and the MCFA for familyIQ dataset is provided in the Appendix. We also performed the equality check for the analytical result of iMCFA with the proposed algorithm. The within-level fixed-effect parameter estimates of target model generated by iMCFA were consistent with Mplus^4^, which is one of the most commonly used SEM software.

In sum, when analyzing complex survey data with level-varying structures, we recommend researchers in the applied areas to use iMCFA to simultaneously perform their analyses with the three proposed modeling techniques. The MAX MCFA model answers research questions about the within-level sampling units, and serves as the baseline for further MCFA construction in response to the level-varying questions for both levels. The factor scores of multilevel measurement analysis from iMCFA could be incorporated in the structure model as the 2-step approach (Anderson and Gerbing, 1988, 1992) to conduct the multilevel SEM analysis. Our illustrations demonstrated that iMCFA can help researchers in their empirical and theoretical study to perform multilevel analyses on complex survey data.

### System requirement, functionality, and future development of iMCFA

iMCFA requires 15 MB of hard disk space to store and has been developed and tested on Windows 7/8/10 32 bits and 64 bits operation systems with LISREL version v8.7 or v8.8 installed. Executing time will vary depending on the complexity of users' model. To consider the computation loading, for the current version of iMCFA, we set the limit of the maximal number of factors as 10, and the maximal number of items as 100.

The iMCFA tool focuses on integrating the functionalities with respect to performing multilevel confirmatory factor analysis with simple or complex structures. By default, iMCFA sets the first indicator of each factor to be the marker variable (e.g., the wordst for f1 and the figure for f2 in Figure 1). Users could re-arrange the input sequence of variables to set the markers. The current version allows only the indicators of continuous scale. Users could set up missing flag in Phase 1 to mark the missingness. To utilize the MUML estimation with multi-group comparison analysis in Lisrel, iMCFA uses the pair-wise deletion for incomplete data to compose level-specific variance-covariance matrices for the complex survey data. With the assumption of Missing at Random (MAR), users can process the incomplete dataset with multiple imputation procedure (Little and Rubin, 1987; Enders, 2010) prior to the use of iMCFA. Users can also revise the generated syntax of three modeling techniques from iMCFA to utilize Full Information Maximum Likelihood method (FIML, Arbuckle, 1996), the default estimation method in Lisrel, for their incomplete raw data with missing values. The equality constraints or fitting multiple-group models are not allowed in the current version of iMCFA.

The function to specify the factor loadings of cross-loaded items and correlated item residuals, and the feature of parameter comparisons with Wald test (Wald, 1943) and family-wise Type-I error rate control among three models will be provided in the following version. In addition, standard errors, *t* values, and significance of corresponding parameters are not included in the iMCFA tabulated output because the output focuses on comparing the model fit of the three modeling techniques. Nonetheless, researchers can use the generated syntax to request the information from LISREL.

Though we applied iMCFA on various types of datasets and models in this study, for more general cases of balanced or unbalanced complex survey data with level-varying structures, the performance of different estimation methods, the sensitivity of level-specific model-fit test statistics and fit indices in detecting lack-of-fit in multilevel CFA and SEM analysis still need more investigation using simulation and empirical approaches.

## Author contributions

J-YW designed the study, conducted the literature review and took a leading role in writing the manuscript. Y-HL helped with data analysis and literature review. JL helped with the programing.

### Conflict of interest statement

The authors declare that the research was conducted in the absence of any commercial or financial relationships that could be construed as a potential conflict of interest. The handling editor is currently editing co-organizing a Research Topic with one of the author JW and confirms the absence of any other collaboration.

## Footnotes

^1^In this study, we refer simple structure CFA as the model with one factor, and complex structure CFA as the model with more than one factors.

^2^Balanced-design samples refer to equal sample size with respect to each group, whereas unbalanced-design samples refer to varying sample sizes in groups.

^3^Users could utilize the generated V-C matrices and the modeling syntax from iMCFA in LISREL v. 9 to request for the analytical result and the diagram. However, due to major changes on the shell commands of LISREL v.9, the current procedure cannot be directly applied on LISREL 9 and its later versions.

^4^In study 2, the simulation dataset was generated in Mplus with FIML estimation. The analytical result were congruent in the within-level fixed-effect estimates between two programs. The noticeable differences between some of the between-level fixed- and random-effects (in Table S.2) would result from the different estimation methods. More simulation studies with comprehensive experimental designs should be conducted to thoroughly investigate the performance of different modeling techniques, estimation methods, and statistical programs on analyzing complex survey data.
