# Supplementary material for: Using iMCFA to Perform the CFA, Multilevel CFA, and Maximum Model for Analyzing Complex Survey Data
Source: Front Psychol. 2018 Mar 13;9:251. doi: 10.3389/fpsyg.2018.00251 (PMC5859678; doi:10.3389/fpsyg.2018.00251)
Supplement: Supplementary file 1 [file DataSheet1.docx]

# Appendix

## Lisrel Syntax for MAX MCFA model

TI: multilevel cfa analysis(max model) between

Use multiple group comparison to perform the MAX MCFA model analysis.

Group: Between group

DA NI=6 NO=60 NG=2 MA=CM

LA

'wordst' 'cards' 'matrix' 'figure' 'animal' 'occpat'

CM

26.88676

19.15107 25.80962

18.06426 17.60424 24.70161

12.12383 11.43514 10.71681 26.42189

14.18866 14.32418 11.71552 18.82468 25.97472

13.86182 13.77826 11.84418 18.50416 19.91530 26.6636

MO NX=6 NK=8 LX=FU,FR PH=SY,FR TD=SY,FR

LK

Set number of different kind of variables and how the factor loadings, covariance between latent variables, and residuals be estimated.

'f1' 'f2'

'B_wordst' 'B_cards' 'B_matrix' 'B_figure' 'B_animal' 'B_occpat'

PA LX

For the lambda x matrix, parameters to be estimated is set to 1 at the corresponding coordinate.

1 0 0 0 0 0 0 0

1 0 0 0 0 0 0 0

1 0 0 0 0 0 0 0

0 1 0 0 0 0 0 0

0 1 0 0 0 0 0 0

0 1 0 0 0 0 0 0

Constrain the factor loadings of the scale

factor to 0.8.

FI LX(1,1) VA 0.800 LX(1,1)

FI LX(4,2) VA 0.800 LX(4,2)

VA 1.000 LX(1,3)

VA 1.000 LX(2,4)

Set factor loadings from the latent variable to the corresponding indicator variable to 1.

VA 1.000 LX(3,5)

VA 1.000 LX(4,6)

VA 1.000 LX(5,7)

VA 1.000 LX(6,8)

ST 0.801 LX(2,1)

Set starting value of the factor loadings at the within level as the values obtained from a single CFA model at the within level.

ST 0.770 LX(3,1)

ST 0.821 LX(5,2)

ST 0.721 LX(6,2)

PA PH

1

1 1

0 0 1

0 0 1 1

For the phi matrix, parameters to be estimated is set to 1 at the corresponding coordinate.

0 0 1 1 1

0 0 1 1 1 1

0 0 1 1 1 1 1

0 0 1 1 1 1 1 1

ST 15.497 PH(1,1)

ST 5.814 PH(2,1)

ST 15.193 PH(2,2)

PA TD

For theta-delta matrix, parameters to be estimated is set to 1 at the corresponding coordinate.

1

0 1

0 0 1

0 0 0 1

0 0 0 0 1

0 0 0 0 0 1

PD

ME=ML: Maximal likelihood estimator.

AD=OFF: No admissibility check.

ND=3: Set number of digits to three.

OU ME=ML AD=OFF ND=3

TI: multilevel cfa analysis(MAX MCFA model) within

Group: Within group

DA NI=6 NO=400 NG=2 MA=CM

CM

16.14617

9.865340 15.28131

9.541270 9.62570 15.59174

3.684660 2.86070 2.84881 16.54743

4.603720 4.42495 3.67369 9.95488 15.09835

3.446340 3.36237 2.73415 8.91399 8.90763 13.25518

MO NX=6 NK=8 LX=IN PH=SY,FR TD=IN

PA PH

1

1 1

0 0 0

0 0 0 0

0 0 0 0 0

0 0 0 0 0 0

0 0 0 0 0 0 0

0 0 0 0 0 0 0 0

EQ LX(1,2,1) LX(2,2,1)

Constrain the parameters at the corresponding location at the within and between level to be equal.

EQ LX(1,3,1) LX(2,3,1)

EQ LX(1,5,2) LX(2,5,2)

EQ LX(1,6,2) LX(2,6,2)

EQ PH(1,1,1) PH(2,1,1)

EQ PH(1,2,1) PH(2,2,1)

EQ PH(1,2,2) PH(2,2,2)

EQ TD(1,1,1) TD(2,1,1)

EQ TD(1,2,2) TD(2,2,2)

EQ TD(1,3,3) TD(2,3,3)

EQ TD(1,4,4) TD(2,4,4)

EQ TD(1,5,5) TD(2,5,5)

EQ TD(1,6,6) TD(2,6,6)

The estimated parameters are saved.

PD

OU ME=ML AD=OFF ND=3

PH=simMCFA_MCFA_MAX_PHI.txt LX=simMCFA_MCFA_MAX_LX.txt TD=simMCFA_MCFA_MAX_TD.txt SV=simMCFA_MCFA_MAX_SV.txt

## Lisrel Syntax for CFA model

Observed Variables:

'wordst' 'cards' 'matrix' 'figure' 'animal' 'occpat'

Sample size= 400

Latent Variables f1 f2

Covariance Matrix

26.88676

19.15107 25.80962

18.06426 17.60424 24.70161d

12.12383 11.43514 10.71681 26.42189

14.18866 14.32418 11.71552 18.82468 25.97472

13.86182 13.77826 11.84418 18.50416 19.91530 26.6636

Relationships

Set up the relations of variables by structural equations.

wordst= 0.8*f1

cards = f1

matrix= f1

figure= 0.8*f2

animal= f2

occpat= f2

LISREL output: ND=3 LX=simMCFA_MCFA_CFA_LX.txt PH=simMCFA_MCFA_CFA_PHI.txt TD=simMCFA_MCFA_CFA_TD.txt SV=simMCFA_MCFA_CFA_SV.txt

Path Diagram

Method of estimation: Maximal Likelihood

End of Problem

## Lisrel Syntax for TWO model

TI MCFA TWO

DA NI=6 NO=400 NG=1 MA=CM

LA

'wordst' 'cards' 'matrix' 'figure' 'animal' 'occpat'

CM

26.88676

19.15107 25.80962

18.06426 17.60424 24.70161

12.12383 11.43514 10.71681 26.42189

14.18866 14.32418 11.71552 18.82468 25.97472

13.86182 13.77826 11.84418 18.50416 19.91530 26.6636

MO NY=6 NE=9 LY=FU,FR PS=SY,FR TE=SY,FR BE=FU,FR

LE

'f1' 'f2' 'B_f1'

'B_wordst' 'B_cards' 'B_matrix' 'B_figure' 'B_animal' 'B_occpat'

PA LY

0 0 0 0 0 0 0 0 0

0 0 0 0 0 0 0 0 0

0 0 0 0 0 0 0 0 0

0 0 0 0 0 0 0 0 0

0 0 0 0 0 0 0 0 0

0 0 0 0 0 0 0 0 0

VA 0.800 LY(1,1)

VA 0.801 LY(2,1)

VA 0.770 LY(3,1)

VA 0.800 LY(4,2)

VA 0.821 LY(5,2)

VA 0.721 LY(6,2)

VA 1.000 LY(1,4)

VA 1.000 LY(2,5)

VA 1.000 LY(3,6)

VA 1.000 LY(4,7)

VA 1.000 LY(5,8)

VA 1.000 LY(6,9)

PA PS

0

0 0

0 0 1

0 0 0 1

0 0 0 0 1

0 0 0 0 0 1

0 0 0 0 0 0 1

0 0 0 0 0 0 0 1

0 0 0 0 0 0 0 0 1

VA 15.497 PS(1,1)

VA 5.814 PS(2,1)

VA 15.193 PS(2,2)

ST 14.445 PS(3,3)

ST 1.496 PS(4,4)

ST 1.099 PS(5,5)

ST 2.138 PS(6,6)

ST 2.001 PS(7,7)

ST 0.790 PS(8,8)

ST 1.619 PS(9,9)

PA TE

0

0 0

0 0 0

0 0 0 0

0 0 0 0 0

0 0 0 0 0 0

VA 6.228 TE(1,1)

VA 0 TE(2,1)

VA 5.335 TE(2,2)

VA 0 TE(3,1)

VA 0 TE(3,2)

VA 6.414 TE(3,3)

VA 0 TE(4,1)

VA 0 TE(4,2)

VA 0 TE(4,3)

VA 6.824 TE(4,4)

VA 0 TE(5,1)

VA 0 TE(5,2)

VA 0 TE(5,3)

VA 0 TE(5,4)

VA 4.859 TE(5,5)

VA 0 TE(6,1)

VA 0 TE(6,2)

VA 0 TE(6,3)

VA 0 TE(6,4)

VA 0 TE(6,5)

VA 5.358 TE(6,6)

PA BE

0 0 0 0 0 0 0 0 0

0 0 0 0 0 0 0 0 0

0 0 0 0 0 0 0 0 0

0 0 0 0 0 0 0 0 0

0 0 1 0 0 0 0 0 0

0 0 1 0 0 0 0 0 0

0 0 1 0 0 0 0 0 0

0 0 1 0 0 0 0 0 0

0 0 1 0 0 0 0 0 0

VA 0.800 BE(4,3)

ST 0.808 BE(5,3)

ST 0.695 BE(6,3)

ST 0.738 BE(7,3)

ST 0.836 BE(8,3)

ST 0.903 BE(9,3)

PD

OU ME=ML AD=OFF ND=3 LY=simMCFA_MCFA_TWO_LY.txt PS=simMCFA_MCFA_TWO_PS.txt TE=simMCFA_MCFA_TWO_TE.txt BE=simMCFA_MCFA_TWO_BE.txt SV=simMCFA_MCFA_TWO_SV.txt

Table S.1 Relative bias of the parameter estimates between Lisrel and Mplus for FamIQ dataset in scenario 1.

*Note.* Relative bias =${(\hat{\theta}_{Mplus}-\hat{\theta}_{Lisrel})}/{\hat{\theta}_{Mplus}}\times100\%$.

Table S.2 Relative bias of parameter estimates between Lisrel and Mplus for simMCFA dataset in scenario 2.

*Note.* Relative bias =${(\hat{\theta}_{Mplus}-\hat{\theta}_{Lisrel})}/{\hat{\theta}_{Mplus}}\times100\%$.
